# Low-level residual viremia and risk of virological failure

**DOI:** 10.1186/1758-2652-13-S4-O5

**Published:** 2010-11-08

**Authors:** G Cologni, L Soavi, S Leone, D Valenti, A Callegaro, G Gregis, F Suter, F Maggiolo

**Affiliations:** 1 Ospedali Riuniti, Bergamo, Italy

## Purpose

The clinical relevance of residual low level viremia (LLV) in patients on steady HAART is debated. Similarly the clinical usefulness of HIV-RNA cut-offs lower than 50 copies/ml is questioned. Aim of this study was to analyze the dynamics of LLV in patients on HAART by means of a high resolution test for HIV-RNA.

## Methods

This is a prospective, single-center, cohort study in patients on stable HAART (mean time on HAART 109 months, SD 28). All patients with a confirmed viremia <50 copies/ml were enrolled. Patients were monitored prospectively with determinations of HIV-RNA every 4 months performed with an enhanced PCR test with a lower limit of detection of 3 copies/ml. ITT analysis is reported.

## Results

A total of 505 patients (78% males) with a mean age of 45.6 years (SD 7.6) were enrolled. At baseline the mean CD4 count was 667 cells/µl (SD 268) and VL was < 3 copies/ml in 73.9% and between 3 and 50 copies/ml in the remaining 26.1% of cases. Over the following 8 months period, patients with a baseline VL < 3 copies/ml presented a stable HIV-RNA below this threshold in 72.8% of cases, a level between 3 and 50 copies/ml in 27.2% of cases while no patient steadily rebounded above the 50 copies/ml threshold. On the contrary, patients with a baseline HIV-RNA between 3 and 50 copies/ml, in the follow-up, presented a stable VL < 3 copies/ml in 43.8% of cases, a VL between 3 and 50 copies/ml in 53.1% of cases, while 3.1% of patients steadily rebounded above the 50 copies/ml threshold (P < 0.0001). In the multivariate analysis the only variable significantly associated with viral dynamics was the third drug in the HAART regimen. A steady VL < 3 copies/ml, a VL between 3 and 50 copies/ml or a steady VL > 50 copies/ml was detected, respectively, in 71.8%; 27.7% and 0.6% of NNRTI-treated patients, while the same figures for PI-treated subjects were 56.2%; 42.9% and 1.0% (P = 0.029).

## Conclusions

The presence of a LLV is associated to a low risk of virological failure; however, in selected patients it may be indicative of effective virus replication leading to virological rebound. Patients treated with a NNRTI-based HAART compared to those receiving a PI-based regimen show a statistically significant more pronounced and steady control of viral replication. Further, prolonged studies are needed to assess the clinical relevance of residual LLV and eventually define new cut-off values predictive of a better control of virus replication.

